# A Case of Internal Hernia Presenting 20 Years After Gastric Bypass

**DOI:** 10.7759/cureus.71570

**Published:** 2024-10-15

**Authors:** Katherine Liu, Brandon Wong, Lyanne Lu, Michael C Larson

**Affiliations:** 1 Radiology, University of California, Davis, Sacramento, USA

**Keywords:** bariatric surgery, internal hernia, mesenteric internal hernia, mesenteric swirl, rou-en-y, roux-en-y complication, roux-en-y gastric bypass (rygb)

## Abstract

Although rare, internal hernias have an increased incidence of development in patients who have undergone Roux-en-Y gastric bypass. They are difficult to diagnose given their non-specific presentation and lack of externally visible findings, and most cases develop within a few months of the original procedure. In this case report, we present a patient with recent weight loss who developed an internal hernia decades post-bariatric surgery with computed tomography findings showing the classic “mesenteric swirl” sign.

## Introduction

In the United States, more than a third of the population is obese [1,2]. Bariatric surgery is well-regarded as one of the most effective treatments, with Roux-en-Y gastric bypass (RYGB) being one of the most common types performed [1,3]. Mesenteric defects formed in the process of creating the bypass predispose patients to develop rare and clinically ambiguous internal hernias [4,5]. If unrecognized, internal hernias can have serious consequences, including incarceration, strangulation, and death [5,6]. We report an internal hernia case with classic findings on computed tomography (CT) in a patient with a remote history of RYGB.

## Case presentation

A 66-year-old woman with a history of suboxone-treated IV drug use, chronic back pain, laparoscopic RYGB, and laparoscopic cholecystectomy presented to the emergency department with two days of intermittent, colicky pain in the upper abdomen with associated nausea, epigastric fullness, and vomiting. During this time, she continued to have normal flatus and regular, formed bowel movements. She denied having previous issues or revisional procedures for her RYGB, which was created using the antecolic technique 20 years ago. Per the patient's report, her pre-procedural weight was 320 lbs, and her post-procedural weight was 220 lbs. However, she also had an unintentional 30 lbs weight loss in the last six months.

Her vital signs at the presentation were stable. On physical exam, her abdomen was soft and distended with epigastric tenderness but without guarding or rebound tenderness. Lab results were unremarkable (Table 1). Abdominal ultrasound was negative for acute pathology. The chest X-ray was also unremarkable aside from a small hiatal hernia at the right cardiac border.

**Table 1:** The patient's lab results at presentation Bolded text are values in the abnormal range. MCV: mean corpuscular volume; MCH: mean corpuscular hemoglobin; MCHC: mean corpuscular hemoglobin concentration; RDW: red cell distribution width; MPV: mean platelet volume; eGFR: estimated glomerular filtration rate

Component	Value	Normal range
Complete blood count		
White blood cell count	9.4	4.5-11.0 k/mm^3^
Red blood cell count	4.71	4.00-5.20 m/mm^3^
Hemoglobin	12.8	12.0-16.0 g/dL
Hematocrit	39.4	36.0-46.0%
MCV	83.8	80.0-100.0 fL
MCH	27.3	27.0-33.0 pg
MCHC	32.5	32.0-36.0%
RDW	14.9	0.0-14.7%
MPV	8.8	6.8-10.0 fL
Platelet count	206	130-400 k/mm^3^
Basic metabolic panel		
Sodium	140	136-145 mmol/L
Potassium	4.3	3.4-5.1 mmol/L
Chloride	103	98-107 mmol/L
Carbon dioxide total	25	22-29 mmol/L
Anion gap	12	7-15 mmol/L
Urea nitrogen, blood (BUN)	15	6-20 mg/dL
Creatinine serum	0.59	0.51-1.17 mg/dL
Glucose	140	74-109 mg/dL
Calcium	9.7	8.6-10.0 mg/dL
e-GFR creatinine (female)	7.4	>30 mL/min/1.73m*2
Urinalysis		
Collection	Clean catch	
Color	Yellow	None/yellow
Clarity	Clear	Clear
pH Urine	5	4.8-7.8
Occult blood urine	Negative	Negative
Bilirubin urine	Negative	Negative
Ketones	80	Negative
Glucose urine	Negative	Negative
Protein urine	Negative	Negative
Urobilinogen	Negative	Negative
Nitrite urine	Negative	Negative
Leukocyte esterase	Negative	Negative
Microscopic	Not indicated	Not indicated
Urine culture	Not indicated	Not indicated
Hepatic function panel		
Protein	7.4	6.6-8.7 g/dL
Albumin	4.3	4.0-4.9 g/dL
Alkaline phosphatase (ALP)	78	35-129 U/L
Aspartate transaminase (AST)	14	≤41 U/L
Bilirubin total	0.5	0.0-0.3 mg/dL
Alanine transferase (ALT)	10	≤33 U/L
Bilirubin direct	0.2	0.0-0.3 mg/dL
Other		
Lipase	14	13-60 U/L
Troponin T	<6	≤19 ng/L
Lactic acid	0.9	0.5-2.2 mmol/L

**Figure 1:**
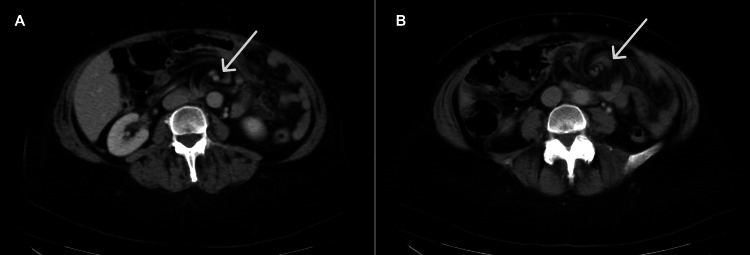
CT abdomen and pelvis (axial). The mesenteric swirl sign, indicative of mesenteric edema and distortion of vessels and bowel loops, can be seen at the arrow in two slices (A: superior; B: inferior)

CT abdomen with contrast showed mesenteric edema stranding and swirling in the left upper abdomen, which was concerning for mesenteric volvulus (Figure 1). Notably, there was a distorted course and caliber of multiple vessels and bowel loops. The superior mesenteric vein was distorted below the pancreaticoduodenal vein with multiple occluded colic branches. There were no definite signs of small bowel obstruction.

The patient was diagnosed with an internal hernia and subsequently taken to surgery for a diagnostic laparoscopy and internal hernia repair. Intraoperatively, the jejunojejunostomy was noted to have twisted on itself, with a portion of the common channel herniated through the jejunojejunostomy mesenteric defect. After untwisting, all segments of the bowel were still viable, and the hernia was reduced with the closure of the defect with a non-absorbable 0-Surgidac running suture. The patient tolerated the procedure well, and she was discharged two days post-operation with no complications.

## Discussion

RYGB patients represent a unique population that is at increased risk of developing internal hernias given their surgically modified anatomy. Internal hernias are rare, with an overall incidence of less than 1% in the general population and between 0.2% and 9% in bariatric surgery patients, but they cause nearly half of all bowel obstructions in post-RYGB patients [5,7]. In present literature, they have been reported to commonly occur 6 to 24 months post-procedure, which is during the period of greatest expected weight loss [8,9]. Weight loss allows defects in the mesentery to reopen despite intraoperative closure, increasing the risk of internal hernias [10]. Our case study exemplifies that there is still a possibility of presentation even as far as 20 years out from the original procedure. In our patient, her risk was likely magnified by her recent, unintentional, and significant weight loss.

The creation of the bypass results in three potential sites of weakness: the transmesocolon mesentery, jejunojejunostomy mesentery, and Petersen’s space [4-8]. A defect in the transmesocolon mesentery is only formed when using the retrocolic technique of joining the Roux limb with the gastric pouch, and these hernias are the most common type [5,7,8,11]. An antecolic technique and surgical standards to take care to close mesenteric defects during the procedure may decrease the overall risk of internal hernias [8,11].

Internal hernias are notoriously difficult to diagnose given their non-specific and variable presentations. Patients with reducible hernias are often asymptomatic, but complications such as strangulation and incarceration can develop and lead to increased mortality if recognition is delayed [5,6]. Common symptoms include epigastric or periumbilical abdominal pain (described as postprandial, vague, or colicky), nausea, and vomiting [5,7,11]. It was reassuring that our patient remained hemodynamically stable and continued to have regular bowel function leading up to the procedure. The absence of laboratory and radiographic signs of bowel obstruction and ischemia further supported a less severe presentation, but surgical treatment was still indicated to minimize further complications.

CT has been the most useful in diagnosis given its ability to offer clarity of anatomic structures compared to plain abdominal radiographs. One of the best predictors is the presence of a “mesenteric swirl” formed from distorted mesenteric fat and vessels [4,7,12,13], as seen in our patient. When seen in conjunction with small bowel obstruction, the sensitivity and specificity can reach up to 96% and 87%, respectively [13]. Other helpful CT signs have been identified, including the mushroom sign, superior mesenteric vein beaking, hurricane eye, criss-cross appearance, small bowel behind superior mesenteric artery, weeping mesentery, and right-sided anastomosis [4,5]. Treatment is surgery to repair the hernia [11].

## Conclusions

We present in this case study a unique internal hernia case that developed 20 years after RYGB, likely complicated by our patient's unexpected weight loss outside of the typical period of post-RYGB weight loss. Internal hernias are notoriously difficult to diagnose given their non-specific presentation and, as exemplified in this case, should be considered in the differential even if patients are years out from their bowel revision surgery. CT is the most reliable modality in work-up, and signs of mesenteric swirling and small bowel obstruction are most predictive of the presence of an internal hernia.
